# Detection and characterization of classical and “uncommon” exon 19 Epidermal Growth Factor Receptor mutations in lung cancer by pyrosequencing

**DOI:** 10.1186/1471-2407-13-114

**Published:** 2013-03-13

**Authors:** Luisella Righi, Alessandra Cuccurullo, Simona Vatrano, Susanna Cappia, Daniela Giachino, Paolo De Giuli, Mara Ardine, Silvia Novello, Marco Volante, Giorgio V Scagliotti, Mauro Papotti

**Affiliations:** 1 Divisions of Pathology, University of Torino, Regione Gonzole 10, Torino, Orbassano, 10043, Italy; 2 Department of Clinical and Biological Sciences, Medical Genetics, University of Torino, Regione Gonzole 10, Torino, Orbassano, 10043, Italy; 3 Pathology Unit, ASLCN2, Cuneo, Alba, Italy; 4 Oncology Unit, ASLTO5, Torino, Carmagnola, Italy; 5 Department of Oncology, Medical Oncology, University of Torino, Regione Gonzole 10, Torino, Orbassano, 10043, Italy

**Keywords:** Lung cancer, EGFR mutation, Exon 19, Pyrosequencing, Adenocarcinoma

## Abstract

**Background:**

The management of advanced stage non-small cell lung cancer is increasingly based on diagnostic and predictive analyses performed mostly on limited amounts of tumor tissue. The evaluation of Epidermal Growth Factor Receptor (EGFR) mutations have emerged as the strongest predictor of response to EGFR-tyrosine kinase inhibitors mainly in patients with adenocarcinoma. Several *EGFR* mutation detection techniques are available, having both sensitivity and specificity issues, being the *Sanger* sequencing technique the reference standard, with the limitation of a relatively high amount of mutated cells needed for the analysis.

**Methods:**

A novel nucleotide dispensation order for pyrosequencing was established allowing the identification and characterization of *EGFR* mutation not definable with commercially and clinically approved kits, and validated in a consecutive series of 321 lung cancer patients (246 biopsies or cytology samples and 75 surgical specimens).

**Results:**

61/321 (19%) mutated cases were detected, 17 (27.9%) in exon 21 and 44 (72.1%) in exon 19, these latter corresponding to 32/44 (72.7%) classical and 12/44 (27.3%) uncommon mutations. Furthermore, a novel, never reported, point mutation, was found, which determined a premature stop codon in the aminoacidic sequence that resulted in a truncated protein in the tyrosine kinase domain, thus impairing the inhibitory effect of specific therapy.

**Conclusions:**

The novel dispensation order allows to detect and characterize both classical and uncommon *EGFR* mutations. Although several phase III studies in genotypically defined groups of patients are already available, further prospective studies assessing the role of uncommon *EGFR* mutations are warranted.

## Background

The old dichotomic distinction between small cell and non-small cell lung cancer (NSCLC) in the last 10 years has been replaced by a more accurate morphological and immunohistochemical subtyping associated to the identification of specific molecular profiles [1,2]. For the management of lung cancer, a crucial issue is the availability of adequate and sufficient tumor tissue not only for pathological diagnosis, but also to allow additional immunohistochemical and molecular studies [3]. Since at diagnosis up to 70% of patients with NSCLC present with inoperable, advanced-stage disease, the histological definition and molecular characterization, including the assessment of the epidermal growth factor receptor (EGFR) sensitizing and resistant mutations, is often based on lung biopsies (endoscopic or transthoracic) or cytological specimens, only. These tumor samples are often characterized by poor cellularity and/or inflammatory or necrotic background containing large amounts of tumor-associated normal cells, which may potentially impair the accuracy of tumor subtyping and molecular characterization [4]. This issue can be improved by the enrichment of tumor cells using tissue microdissection prior to mutational analysis [5].

The detection of activating *EGFR* mutations is nowadays the best predictive marker to treat NSCLC with EGFR-Tyrosine Kinase Inhibitors (TKI) [6], but most trials conducted so far are based on a limited number of known *EGFR* mutations, including the point mutation at codon 858 of exon 21 (NM_005228.3 p.Leu858Arg) and the numerous in-frame deletions in exon 19, which account for more than 90% of mutations [7]. Furthermore, a single, standardized method to perform the mutational analysis is not yet available [8], making rigorous quality control tests mandatory for each laboratory. Numerous methodological approaches are currently available although affected by great inter- and intra-laboratory variability in terms of performance and lack of adequate quality controls. The use of commercial kits certified by the FDA and/or EMEA is therefore recommended in the clinical practice [9]. The most commonly used mutation detection techniques (i.e. *Sanger* sequencing, Pyrosequencing, HRMA - High Resolution Melting Analysis and ARMS – Amplification Refractory Mutation System analysis) were established to offer sensitive molecular analysis, all including DNA extraction, PCR amplification and subsequent genetic test. Thus far, the *Sanger* DNA sequencing method is the reference method used for the detection and identification of *EGFR* mutations in tumor cells, because it provides the exact nucleotide sequence of the segment amplified, despite its sensitivity is lower than others, especially in the case of small tumor samples, since it requires at least 50% of mutated tumor cells [10], corresponding to 20–25% of mutated DNA in an heterozygous case [8]. More recently, several assays have been developed to improve mutation detection in terms of sensitivity (to better perform molecular analyses even in very small specimens) and also of specificity (to recognize and characterize multiple mutations at the same time). Indeed recently, beyond the classical therapy-responsive mutations, some “uncommon” mutations were described whose clinical significance is still poorly understood [11].

Pyrosequencing is a DNA sequencing technology “by synthesis” with luminometric detection. Due to its modalities of analysis and nucleotide dispensation [12], any change from normal in the target sequence is detected as a pyrogram alteration, but not characterized unless corresponding to an expected genetic alteration [13]. Furthermore, the commercially available pyrosequencing kit properly identifies commonest *EGFR* point mutations (i.e. exons 18 NM_005228.3 p.Gly719Ser, p.Gly719Cys, p.Gly719Ala, p.Gly719Asp and exon 21 NM_005228.3 p.Leu858Arg, p.Leu861Gln), but is certified to detect just a positive mutational status in the presence of the two most frequent (classical) deletions in exon 19 (NM_005228.3 c.2235_2249del15 and c.2236_2250del15 - p.Glu746_Ala750del). On the contrary, all the other uncommon exon 19 mutations are detected as an altered signal, although not further identifiable.

The aim of the present study was to improve the performance of pyrosequencing assay for *EGFR* mutation detection by setting up a novel dispensation order (NDO) capable not only to detect but also to characterize the type of mutations of exon 19 associated to the responsiveness to TKI therapy [14], and to validate its efficacy in the clinical setting in a consecutive prospectively collected series of lung cancer specimens.

## Methods

### Cell lines and plasmids

The human lung cancer HCC827 and H522 cell lines were obtained from the American Type Culture Collection and were cultured in RPMI 1640 supplemented with 10% fetal bovine serum at 37°C in air containing 5% CO_2_. The HCC827 cell line harboured in homozygosis one of the two classical *EGFR* deletions in the tyrosine kinase (TK) domain (NM_005228.3 p.Glu746_Ala750del, c.2236_2250del15), while the control H522 cell line was wild type (*wt*) for exon 19 mutations. DNA from H522 was used to dilute the mutated HCC827 DNA cell line at 50% and 25% of mutated allele in order to test the NDO accuracy to detect the presence of the classical *EGFR* deletions.

Furthermore, to test the sensitivity of NDO detection (set as the minimum percentage of mutation detection), the DNA plasmid pUC57 (Eurogentec, Belgium, kindly purchased by Diatech Company - Jesi, Italy), containing an insertion of 400 bp harbouring three different in-frame deletions of exon 19 *EGFR* in homozygosis (the other classical NM_005228.3 p.Glu746_Ala750del, c.2236_2250del15 (M1882), and the uncommon c.2240_2257del18 (M1883) in-frame deletion and c.2239_2248delinsC (M1884) complex mutation) was mixed with the *wt* plasmid M1880 at serial descending dilutions, obtaining 50%, 25%, 12.5% and 6.25% of exon 19 mutated DNA.

### Tissue samples

From January 2010 to December 2011, 334 consecutive NSCLC samples (including 75 surgical resections, 139 transthoracic or endoscopic biopsies and 96 cytological specimens) were considered for *EGFR* (GenBank NM_005228.3) exons 18, 19, 21 mutational analysis in the Pathology Division of the University of Turin at San Luigi Hospital, and subsequently prospectively collected for NDO pyrosequencing analysis. A pathologist (LR) evaluated the adequacy of all cases selecting the tissue specimens having the highest tumor cell content. Adequacy was set at a minimum of 200 tumor cells and a percentage of tumor cells in the DNA sample of at least 30%. Such adequacy assessment is in line to current national and international recommendations/guidelines [9,15]. Enrichment of tumor cells was obtained by manual microdissection under light microscopy from one to ten sections for each case. Briefly, 5 μm thick sections of tumor samples were collected on glass slides and processed with a fast haematoxylin-eosin staining. Tumoral cells were scraped with a 1 mm-gauge needle in 70% ethanol and collected in vials. After centrifugation at 14000 rpm for 20 minutes, the microdissected pellet obtained for each sample was dehydrated with absolute ethanol, followed by another centrifugation at 14000 rpm for 20 minutes. The dried pellets were processed for DNA extraction.

All histological material was de-identified and cases were anonymized by a pathology staff member not involved in the study. Clinical data were compared and analysed through coded data, only. The study was approved by the institutional review board of San Luigi Hospital, Turin, Italy.

### DNA extraction and PCR amplification

Genomic DNA from formalin-fixed paraffin-embedded (FFPE) cell lines and tissues was extracted and purified using *QIAmp DNA FFPE Tissue* kit (Qiagen, Hilden,Germany) specific for purification from FFPE samples, according to the manufacturer’s instructions. The amount of DNA obtained was quantified by spectrophotometry (Eppendorf, Hamburg, Germany). Genomic DNA from cell lines, tissues and plasmid was amplified by real-time end-point PCR using *EGFR TKI response (sensitivity)* kit (CE-IVD, Diatech, Jesi, Italy) according to the manufacturer’s instructions, using *Rotor-Gene Q* (Qiagen, Hilden, Germany). After amplification, the presence of PCR products was detected by melting-analysis with a denaturation step from 65°C up to 95°C. The specific melting temperatures of exon 18, 19 and 21 amplicons were 84.5°C, 81.3°C and 83°C, respectively. Wild type and no template samples were added in each assay as positive and negative controls.

### Mutational analysis by pyrosequencing and novel nucleotide dispensation order

The mutational analysis was performed by pyrosequencing with PyroMark Q96MA apparatus (Biotage, Uppsala, Sweden) using *EGFR TKI response (sensitivity)* kit. PCR and mutational analysis were conducted in duplicate for each sample, as requested by the guidelines. Only the genomic regions frequently harbouring mutations relevant for TKI therapy [14] were analyzed (i.e. for ex 18: codons from 2149 to 2157; for ex 21: codons from 2572 to 2585; for ex 19: codons from 2234 to 2250). The pyrograms obtained were analysed following the manufacturer’s instructions.

**Figure 1:**
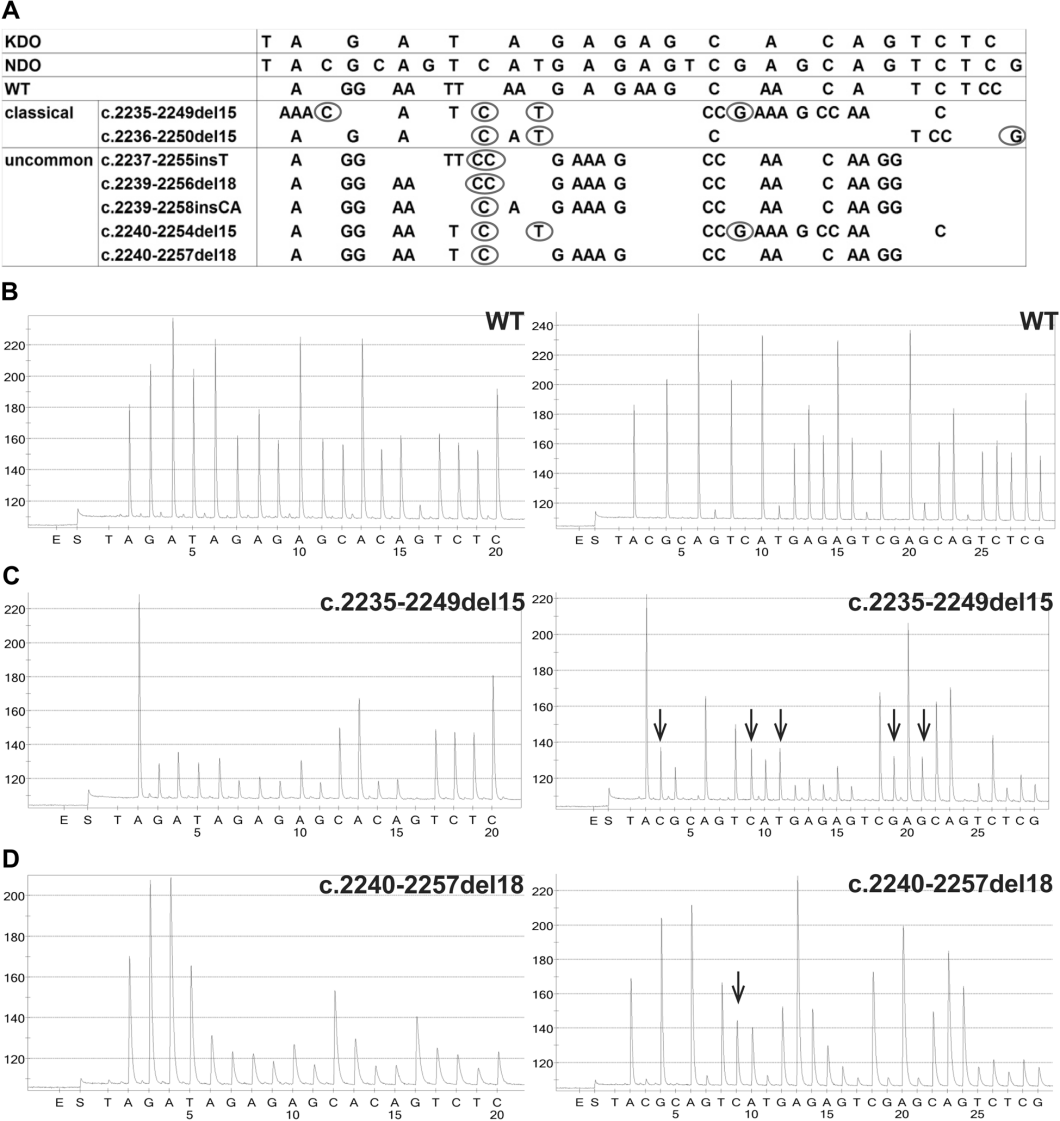
**Nucleotide sequences and representative pyrograms of the commercial kit and novel dispensation order for EGFR exon 19 mutation analysis.** (**A**) In the home-made dispensation order a number of nucleotides major than commercial is present to characterize a wider group of deleted sequences (classical and uncommon) than commercial kit. Circles exemplify anomalous nucleotides identified by NDO only. Example of pyrograms obtained using the commercial KDO (left panels) and NDO (right panels) for pyrosequencing in samples with EGFR exon 19 (**B**) wild type, (**C**) classical deletion (c.2235-2249del15), (**D**) uncommon deletion (c.2240-2257del18). Arrows indicate peak modifications corresponding to anomalous nucleotides identified by NDO only (Abbreviations: KDO: kit dispensation order; NDO: novel dispensation order; WT: wild type).

To better characterize both common and uncommon mutations affecting *EGFR* exon 19 in the studied genomic region, PCR primers generating an amplimer of 113 bp (sense- 5^′^-TCCCAGAAGGTGAGAAAGTTAAA-3^′^ and antisense BIO-5^′^-CCACACAGCAAAGCAGAAAC-3^′^) and a sequencing primer (5^′^-TTCCCGTCGCTATCA-3^′^) were designed using the PSQ Assay Design software (Biotage) and a novel NDO (5^′^-TACGCAGTCATGAGAGTCGAGCAGTCTCG-3^′^) for pyrosequencing was developed analyzing the codons from 2234 to 2259. The NDO consisted in a sequence of 29 dispensed nucleotides, longer than commercial kit dispensation order, in which additional bases are introduced in strategic positions, according to possible expected mutated sequences (Figure 1A). The obtained pyrograms allowed to characterize the sequences different from *wt* resulting from either classical or uncommon mutations (Figure 1B, C, D).

### Mutational analysis by ARMS

To validate the results obtained by pyrosequancing, as indicated by guidelines [9], all tumor samples were also tested for the presence of the two most frequent *EGFR* exon 19 deletions (NM_005228.3 c.2235_2249del15 and c.2236_2250del15, p.Glu746_Ala750del) by ARMS using the following primers: sense 5^′^-TGCATCGCTGGTAACAT-3^′^ and antisense 5^′^-CGGAGATGTTTTGATAGCG-3^′^[16]. Briefly 25 μl of PCR reaction mix contained 2X Master mix (Promega, Madison - USA), 20X EVA Green dye (Biotium, Hayward, CA - USA), 0.6 μM of each primer and 50 ng DNA template. PCR conditions on *Rotor-Gene Q* (Qiagen Hilden, Germany) were as follows: 95°C for 5 minutes, then 40 cycles of 95°C for 30 seconds, 56.6°C for 30 seconds and 72°C for 30 seconds, followed by a melting profile with a ramping range of 65° to 95°C and rising steps of 1 degree. Positive and no template controls were added to each tests.

### Sanger sequencing analysis

In selected tissue cases, *EGFR* exon 19 uncommon mutation were also confirmed by di-deoxy *Sanger* direct sequencing analysis, supported by Eurofins MWG Operon service, (Ebersberg, Germany), after DNA amplification. Briefly, the target region was amplified with sense 5^′^-ACAATTGCCAGTTAACGTCTTCCT-3^′^ and antisense 5^′^-ATGAGAAAAGGTGGGCCTGA-3^′^ primers under the following conditions: 95°C for 5 minutes, then 40 cycles of 95°C for 30 seconds, 55°C for 40 seconds and 72°C for 40 seconds, followed by 72°C for 4 minutes. Then, the PCR products were resolved by 2% agarose gel electrophoresis to confirm successful amplification and purified using *MicroSpin*™ S-400 HR Columns kit (GE Healthcare, Buckinghamshire, UK), according to the manufacturer’s instructions.

### Statistical analysis

Distribution of *EGFR* mutations as compared to patients’ variables were analyzed with One-way ANOVA test and Fisher’s test using GraphPad Prism software, version 5.0. The level of significance was set at *p* < 0.05.

## Results

### Validation of the novel dispensation order on cell lines and plasmids

On cell lines, the *EGFR TKI response (sensitivity)* kit and the NDO procedure were both able to detect the classical deletion in the HCC827 DNA cell line at the higher (50%) and lower (25%) dilutions in terms of peak profile alteration, whereas EGFR *wt* DNA cell line resulted in the normal profile.

In the analysis of DNA plasmid with the *EGFR TKI response (sensitivity),* the classical mutation was detected up to 25% of dilution, while uncommon mutations were reported as indeterminate or *wt* when the dilution reached the lowest percentages of mutated alleles. On the contrary, the NDO showed specific peak alterations corresponding to the three different deletions on plasmid allowing to characterize all mutations up to 6.25% of mutant allele dilution.

### Mutational analysis on tissue samples

Thirteen cases (3.8%) were excluded because not reaching the adequacy standards set for molecular analysis, based on national and international recommendations [9,15] even after tumor cell enrichment by manual microdissection.

The study samples derived from 186 men (57.9%) and 135 women (42.1%), with a median age of 65 years (range 16 to 89 years); specimens were represented by 75 (23.4%) primary lung tumor resections and 246 (76.6%) non-surgical (150 endoscopic transthoracic or lymph node biopsies and 96 cytological) samples; 226 (70.4%) tissues were from pulmonary tumor location and 95 (29.6%) were metastases. The final diagnoses included: 269 (83.8%) adenocarcinomas (ADC), 37 (11.6%) NSCLC, favor ADC, four (1.2%) NSCLC not otherwise specified and 11 (3.4%) non-ADC. In particular, surgical case series included: 69 ADC and 6 non-ADC (2 squamous cell carcinoma; 2 large cell carcinoma, 1 mucoepidermoid low grade carcinoma and 1 sarcomatoid carcinoma having 80% of ADC component). Non-surgical case series corresponded to 200 cases with a morphology-only based ADC diagnosis, 37 NSCLC favouring ADC after immunohistochemistry (IHC), four NSCLC not otherwise specified for an ambiguous immunophenotype and five non-ADC cases (4 squamous and 1 undifferentiated carcinoma). Patients’ characteristics are summarised in Table 1.

**Table 1:** **Case series characteristics and*EGFR*mutations distribution**

	***N***	***EGFR *mutations**			
		**Total mutated**	***p *value**	**EXON 19**	**EXON 21**
**Total**	321	61 (19.0%)		44 (72.1%)	17 (27.9%)
**Sex**					
Male	186 (57.9%)	21 (11.3%)	<0.0001	14 (66.7%)	7 (33.3%)
Female	135 (42.1%)	40 (29.6%)		30 (75.0%)	10 (25.0%)
**Surgical samples**	75 (23.4%)	17 (22.6%)	*0.40*	10 (58.8%)	7 (41.2%)
**Non surgical samples**	246 (76.6%)	44 (17.8%)		34 (77.2%)	10 (22.7%)
Biopsies	150 (46.7%)	26 (17.3%)	*0.86*	20 (76.9%)	6 (23.1%)
Cytology (cell blocks)	87 (27.1%)	16 (18.3%)		14 (87.5%)	2 (12.5%)
Cytology (smears)	9 (2.8%)	2 (22.2%)		0	2 (100%)
**Location**					
Primary tumors	226 (70.4%)	45 (19.9%)	*0.64*	33 (73.3%)	12 (26.7%)
Metastases	95 (29.6%)	16 (16.8%)		11 (68.8%)	5 (31.2%)
**Diagnosis**					
ADC	269 (83.8%)	53 (19.7%)	*0.83*	37 (69.8%)	16 (30.2%)
NSCLC (*favor* ADC)	37 (11.6%)	7 (18.9%)		7 (100%)	0 (0%)
NSCLC NOS	4 (1.2%)	0		-	-
Non-ADC	11 (3.4%)	1 (9.1%)		0 (0%)	1 (100%)

Legend: *ADC*: adenocarcinoma; *NSCLC*: non small cell lung cancer; *NOS*: non otherwise specified.

*EGFR* mutational status was distributed as follows: total mutated cases were 61/321 (19.0%), including 17/61 (27.9%) in exon 21 and 44/61 (72.1%) in exon 19. Among surgical samples, 17/75 (22.6%) mutated cases were found, including 10/17 (58.8%) mutations in exon 19 and 7/17 (41.2%) mutations in exon 21 (all p.L858R type). In non-surgical samples, 44/246 (17.8%) mutated cases were found, of which 34/44 (77.2%) mutations were in *EGFR* exon 19 and 10/44 (22.7%) point mutations in exon 21 (9 p.L858R and 1 p.L861Q). As to concern primary pulmonary versus metastatic tumor samples, mutations were detected in 45/226 (19.9%) and 16/95 (16.8%) cases, respectively. Moreover, distribution of mutated cases according to diagnosis was 53/269 (19.7%) ADCs, 7/37 (18.9%) NSCLC favour ADC and 1/11 (9.1%) non-ADC (sarcomatoid carcinoma). No mutations in exon 18 were found.

The distribution of *EGFR* mutations among male and female patients was significantly different: the percentage of mutations in women was nearly double than that of male patients (Fisher test, p < 0.0001). On the contrary, no significant differences were recorded as compared to other characteristics, including type of sample (surgical, biopsy or cytology) (Table 1).

**Figure 2:**
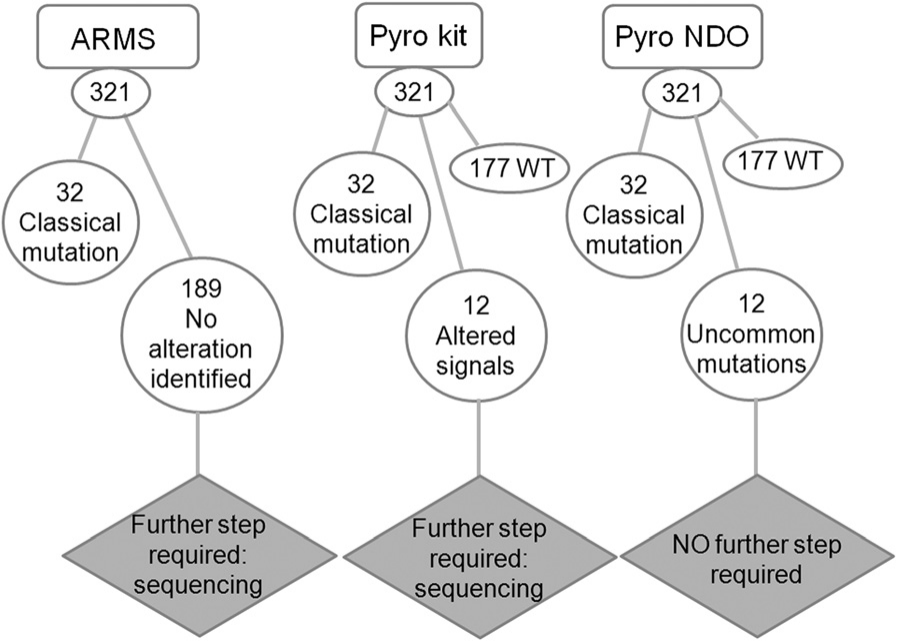
***EGFR *exon 19 mutational analysis on 321 prospectively collected samples.** The diagram illustrates the results obtained using ARMS technique, *EGFR TKI response (sensitivity)* kit for pyrosequencing and the home-made dispensation order for *EGFR* exon 19 mutational analysis. As compared to *EGFR TKI response (sensitivity)* kit, the NDO allowed to characterize the specific nucleotidic change in the presence of uncommon mutations in a single step, avoiding further need of *Sanger* sequencing. (**Abbreviations**: Pyro-kit: commercial pyrosequencing analysis; Pyro-NDO: novel dispensation order for pyrosequencing; *wt*: wild type; ARMS: Amplification Refractory Mutation System; mut: mutations).

The *EGFR TKI response (sensitivity)* commercial kit was able to detect all mutated cases as an altered signal with respect to wild-type sequence either in exon 21 and exon 19; of these latter 32/44 (72.7%) were attributable to one of the two classical more common in-frame deletions, while 12/44 (27.2%) were referred as altered signals by the kit with no possibility of further characterization. On the other hand, ARMS analysis was able to confirm all samples (32/44 cases) harboring the classical exon 19 deletions with a specific amplification. Nevertheless the remaining samples resulted negative because did not show amplification, included not only the true *wt* cases but also those cases harboring uncommon mutations in exon 19. No amplification was detected in no-template controls. On the contrary, using the above described newly constructed NDO, it was possible not only to detect all the 61/321 alterations (with a 100% concordance with *EGFR TKI response* commercial kit), but also to exactly characterize both the 44/61 classical and the 12/61 uncommon mutations of exon 19, with a similar performance on cytological and histological material (Figure 2). The uncommon mutations identified by NDO were subsequently sequenced by *Sanger* method for confirmation only, although this latter procedure would not be necessary to determine the type of mutation and could be avoided in the clinical practice.

**Figure 3:**
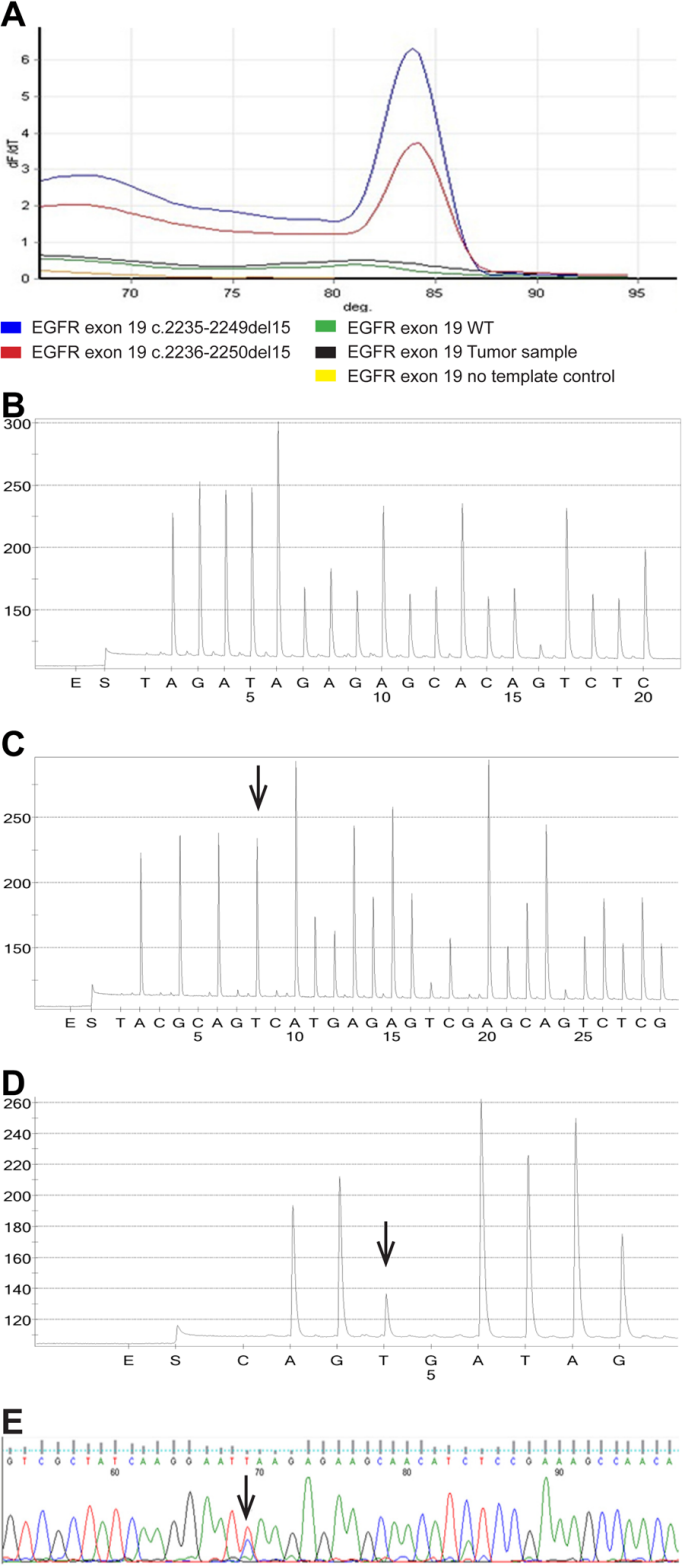
**Sequence analysis of the tumor sample harbouring the novel mutation in EGFR exon 19.** (**A**) ARMS analysis: the amplification of EGFR exon 19 is positive in the cases harbouring the two classical deletions (c.2235-2249del15 and c.2236-2250del15) in comparison to negative *wt* sample, no template control and the test tumor sample harbouring the new mutation. (**B**). *EGFR TKI response (sensitivity)* kit sequencing using commercial dispensation order: the pyrogram is altered but not referable to any classical or uncommon mutation. (**C**). *EGFR TKI response (sensitivity)* kit sequencing using NDO: the pyrogram allows to determine a suspected substitution (arrow) of a new nucleotide. (**D**) Pyrogram trace of the sample obtained using a specific nucleotide dispensation order allows to characterize the new mutation as an insertion of T nucleotide (arrow) in the place of a G nucleotide. (**E**) *Sanger* confirmation of the new mutation identified (arrow).

Moreover, using this approach, a new mutation, which had never been described in the literature nor reported in any database of genetic variants (http://www.sanger.ac.uk/genetics/CGP/cosmic/; http://www.ncbi.nlm.nih.gov/dbvar/; http://www.uniprot.org/uniprot/P00533#ref83; http://www.ensembl.org/Homo_sapiens/Search/Details?db=core;end=498;idx=Somatic_mutation;q=egfr; species = Homo_sapiens), was identified in exon 19. This occurred in a lung ADC case that showed no alteration at ARMS analysis (Figure 3A), an altered pyrogram with the *EGFR TKI response (sensitivity)* kit (Figure 3B) but not attributable neither to classical or uncommon mutations affecting *EGFR* exon 19 so far described. On the contrary, with the NDO mutational analysis a nucleotide substitution was hypothesized (Figure 3C). Subsequently, we developed a further dispensation order (5^′^-CAGTGATAG-3^′^) based on the hypothesized nucleotidic change capable to better characterize the alteration identified. The new, more specific pyrogram obtained showed the presence of a point mutation, c.2236 G > T (ENST275493; NM_005228.3) (p.Glu749* - ENSP00000275493; NP_005219.2; P00533) (Figure 3D), further confirmed by dideoxy *Sanger* sequencing (Figure 3E). This mutation occurred in the coding region of the Tyrosine Kinase protein domain (from aa 712 to aa 968) of the EGFR receptor (P00533_http://www.uniprot.org/) and generates a premature stop-codon possibly leading to a shorter transcript and subsequently to the formation of a truncated protein, possibly causing the lack of a part of the Tyrosine Kinase domain and of the C-terminal portion (from aa 749 to aa 1210). *In silico* studies using bioinformatic simulations (http://www.fruitfly.org/seq_tools/splice.html; http://bioservices.usd.edu/splicepredictor/; http://www.cbs.dtu.dk/services/NetGene2/; http://www.itb.cnr.it/sun/webgene/) performed to evaluate the possible effects of this mutation on primary transcript splicing didn’t show any possible significant modification.

## Discussion

In the present study, we performed *EGFR* mutational analysis in a prospectively collected NSCLC sample series with pyrosequencing technique and we further constructed an home-made dispensation order for pyrosequencing that allowed not only to recognize in a single step but also to characterize both the classical and the uncommon mutations affecting exon 19 in the region from codon 2234 to 2259, that is the region most frequently affected by mutations relevant for TKI therapy [14], The analysis was informative of *EGFR* mutational status in more than 95% cases of NSCLC, while non-eligible specimens were mainly cytological material with a very low amount of neoplastic cells. For the remaining samples, when the tumor tissue was present only in a small fraction of the biopsy and/or dense inflammatory infiltrates were detected, the preliminary procedure of microscope-assisted manual microdissection and sample enrichment for neoplastic cells allowed to obtain at least 100 tumor cells and all samples could successfully be analysed for *EGFR* mutations [8,17].

The interest in the mutational analysis on small cytological samples increased in recent years [4] and several studies are ongoing with different techniques to develop new guidelines [2]. In our study, no difference in term of mutation detection was found between surgical and non-surgical samples; furthermore, among non-surgical, none of different fixation methods (formalin for biopsies and alcohol for cytology) impaired the mutation detection.

A similar detection rate was observed in tumor samples from primary and metastatic locations thus confirming the same distribution of *EGFR* mutations within the primary tumor and between primary tumor and metastases that are still matters of debate [18,19]. Recent studies found high disease control rate even though small biopsy or cytology specimens were a source for EGFR test [20,21]. Thus, testing of small cytological or biopsy samples from the primary site or metastases may be representative of the whole carcinoma genotype [8].

Furthermore, no difference in mutation distribution was found between morphologically determined ADC and NSCLC favouring ADC after IHC [6], thus confirming that the histotyping of NSCLC non otherwise specified is important to select patients for mutational analysis.

In this study, in agreement with data from others [22], the majority of *EGFR* mutations were in-frame deletions in exon 19. Furthermore, a slightly higher amount of mutated cases compared to the literature data in the Western population [23] was observed, and this could be attributed to the high sensitivity of pyrosequencing. Such results were confirmed either with ARMS assay, that demonstrated a good concordance though providing information only in terms of presence/absence of classical mutations (with no information about any other alteration), and with *Sanger* sequencing that provides the exact nucleotide sequence (though with a lower sensitivity [10]). Recent technological advances enabled the development of several more sensitive and rapid methods than direct sequencing for the detection of *EGFR* mutations in multiple biological samples. These methods (including LNA/PNA clamp, TheraScreen, SNAPshot PCR procedures) are able to detect mutant alleles occurring at frequencies as low as 0.1%, but the results obtained are restricted to a screening of mutant versus wild type tumors, in the lack of any further characterization, as currently required [9,24]. Pyrosequencing performs well on shorter DNA sequences than *Sanger* sequencing (for this reason it represents one of the most sensitive methods in those cases associated to poor cellularity because of small sample size and/or to potentially damaged DNA). Nevertheless, the commercial *EGFR TKI response (sensitivity)* kit for pyrosequencing was able to detect all abnormal cases, but not to characterize *EGFR* mutations other than the two most common in-frame deletions (NM_005228.3 c.2235_2249del15 and c.2236_2250del15 - p.Glu746_Ala750del). Thus, in the presence of an uncommon mutation occurring in the studied sequence for TKI therapy, it is difficult, and somehow arbitrary, to associate the “atypical” pyrogram profile with the corresponding mutation. Furthermore, in case of an unknown, never described, mutation (deletion, insertion or any other type) that occurred in the same analyzed sequence, the pyrogram will be altered with respect to the wild type, but with no way to describe the exact mutation. To overcome this limitation, we re-sequenced all cases with the above described home-made NDO for pyrosequencing, allowing to better define the 12/44 (27.2%) uncommon mutations in exon 19, occurring in the region from codon 2234 to 2259.

Moreover, we further identified a new uncommon mutation, a point mutation occurred in the Exon 19 (c.2236 G > T ENST275493; NM_005228.3) not possible to be characterized by the conventional EGFR kit, resulting by means of NDO in a transversion possibly leading to an early stop codon and to the synthesis of a truncated EGFR protein lacking of a part of the Tyrosine Kinase domain and also of the near C-terminal portion. Such mutation was also confirmed by *Sanger* sequencing. The lost portion of the protein is probably the most essential for its intracellular function, therefore its almost total absence might lead to a signal transduction interruption and to an inactive EGFR protein, thus rendering useless the TKI drug administration. As a matter of fact, the patient failed to respond to the TKI therapy, although functional studies are necessary to better clarify the patho-physiological implications of this particular mutation. The significance of uncommon mutations is uncertain. In fact, on the one side it is well known that care must be taken when working with small amounts of DNA (e.g. from FFPE biopsies) to avoid artifactual mutation detection [25,26]. In this respect, large amounts of template DNA were used and multiple amplifications examined, as recommended [17]. On the other side, the clinical relevance of rare mutations is necessarily to be linked with response to specific treatment. Some lung cancer patients harbouring never described mutations and experiencing an unexpected response to gefitinib have already been reported [11]. By contrast, other rare mutations such as the insertion in exon 19 recently described by Otto and co-workers [27], which is not recognizable by our actual NDO protocol, needs to be further validated in the clinical practice as markers of responsiveness to TKI therapy. For this reason, new information are expected from clinical and outcome data on patient bearing uncommon *EGFR* mutations [7], as well as the results of trials with EGFR inhibitors designed introducing common versus uncommon mutations as stratification factor in the randomization schema [28].

In our series, clinical outcome data on 26/44 exon 19 mutated patients who underwent second- or third-line therapy with gefitinib were available, including 20/26 classical and 6/26 uncommon deletions. Among the six (of 26) responsive patients, 3/20 (15%) bore classical and 3/6 (50%) had uncommon deletions. These preliminary data may indicate that uncommon mutations in general are more probably associated to sensitivity rather than resistance to therapy.

## Conclusions

Our results overall strengthen the overwhelming necessity of cost-effective and practical methods for *EGFR* mutation detailed characterization [7] for NSCLC patient management, even when dealing with small amount of tumoral tissue. Correlative studies comparing each type of *EGFR* mutation with specific clinical response to EGFR inhibitors are necessary, with special attention to poorly responding patients.

## Abbreviations

NSCLC: Non small cell lung cancer; EGFR: Epidermal Growth Factor Receptor; HRMA: High resolution melting analysis; ARMS: Amplification refractory mutation system; NDO: Novel dispensation order; TK: Tyrosine Kinase; FFPE: Formalin-fixed paraffin-embedded; TKI: Tyrosine kinase inhibitor; ADC: Adenocarcinoma; IHC: Immunohistochemistry.

## Competing interests

GVS: honoraria from Eli-Lilly, Astra-Zeneca, Roche, Pfizer. All the other authors have no competing interest to declare with regard to the topic covered in this study.

## Authors’ contributions

LR, SC: have made substantial contributions to conception and design, to analysis and interpretation of data and have been involved in drafting the manuscript; AC, VS, DG: have made substantial contributions to conception and design and to acquisition and analysis of data; PDG, MA, SN: have made contributions to acquisition of data, MV, MP, GVS: have been involved in revising it critically for important intellectual content and have given final approval of the version to be published. All authors read and approved the final manuscript.

## Authors’ information

CA is recipient of a scholarship supported by Fondazione Elena e Gabriella Miroglio, ONLUS, Alba (Cuneo). VS is a graduate student of PhD program, University of Turin.

## Pre-publication history

The pre-publication history for this paper can be accessed here:

http://www.biomedcentral.com/1471-2407/13/114/prepub
